# BCL11A Promotes the Progression of Laryngeal Squamous Cell Carcinoma

**DOI:** 10.3389/fonc.2020.00375

**Published:** 2020-03-20

**Authors:** Jian Zhou, Liang Zhou, Duo Zhang, Wei-Jing Tang, Di Tang, Xiao-Ling Shi, Yue Yang, Lin Zhou, Fei Liu, Yong Yu, Pentao Liu, Lei Tao, Li-Ming Lu

**Affiliations:** ^1^Shanghai Institute of Immunology, Shanghai Jiaotong University School of Medicine, Shanghai, China; ^2^Department of Otolaryngology, Eye, Ear, Nose and Throat Hospital, Fudan University, Shanghai, China; ^3^Shanghai Key Clinical Disciplines of Otorhinolaryngology, Shanghai, China; ^4^Wellcome Trust Sanger Institute, Cambridge, United Kingdom

**Keywords:** BCL11A, LSCC, proto-oncogene, MDM2, prognosis

## Abstract

**Background:** We report functional and clinical data uncovering the significance of B-cell lymphoma/leukemia 11A (BCL11A) in laryngeal squamous cell carcinoma (LSCC).

**Methods:** We examined BCL11A expression in a cohort of LSCC patients and evaluated the association between BCL11A expression and clinicopathological features. We investigated the consequences of overexpressing BCL11A in the LSCC cell line on proliferation, migration, invasion, cell cycle, chemosensitivity, and growth *in vivo*. We explored the relationship between BCL11A and MDM2 in LSCC and tumorigenesis pathways by using the Human Cancer PathwayFinder Array.

**Results:** High levels of BCL11A were found in LSCC tissues and were more frequently associated with advanced lymphatic metastasis stages with poor prognoses. BCL11A overexpression enhanced LSCC proliferation *in vitro* and *vivo*. A positive correlation between MDM2 and BCL11A expression was identified.

**Conclusions:** These data uncover important functions of BCL11A in LSCC and identify BCL11A as a prognostic biomarker and potential therapeutic target in LSCC.

## Introduction

Laryngeal squamous cell carcinoma (LSCC) is the eleventh most common tumor in males. The most deep-rooted risk factors for LSCC are alcohol abuse and tobacco use (1). The LSCC has a poor prognosis, with a 5 year survival rate <50%. The aforementioned is mostly attributed to regional and local recurrences, especially in patients with stage III or IV disease (2). Studies are needed to increase our understanding of the pathogenesis and prognosis of LSCC.

The *Bcl11a* gene encodes a C2H2 zinc finger transcription factor, which acts as a retroviral insertion site (Evi9) in myeloid leukemia in BXH-2 mice (3, 4). The gene was initially recognized in a rare *t*_(2;14)_ (p16; q32.3) translocation in aggressive B-cell chronic lymphocytic leukemia, and it is usually co-amplified with the proto-oncogene REL in classical Hodgkin's lymphoma and non-Hodgkin's lymphoma (5, 6). The role of BCL11A in solid melanomas has not been widely studied. Khaled et al. studied the BCL11A oncogene in triple-negative breast tumors and reported that its overexpression promoted cancer development (7). Jiang et al. revealed that BCL11A protein expression levels were markedly upregulated in non-small-cell lung cancer (NSCLC) tissues, particularly in large cell cancer and squamous cell cancer (SCC). A multivariate analysis demonstrated that BCL11A was an independent prognostic factor for both overall survival and disease-free survival (8). To address these issues, we determined BCL11A expression in LSCC and investigated its clinical significance by associating clinicopathologic parameters with BCL11A expression in LSCC patients. We further dissected the cellular and molecular mechanisms underlying BCL11A function in LSCC.

## Materials and Methods

### Patient Population

Sixty-nine LSCC patients were recruited for this study. All of them had undergone total laryngectomy, with or without chemotherapy from November 2013 to September 2014 in the Otolaryngology Head and Neck Surgery Department of the Eye, Ear, Nose and Throat Hospital, Fudan University. Of 69 patients entered in this research, suitable tissues for Tissue Microarrays (TMA) were available for all 69 patients. All patients were categorized on the basis of UICC stage classification using specimens. All cases were collected according to study protocols permitted by the Ethics Committee of Fudan University, and all the participants offered written informed consent before the commencement of the research. The following data were recorded: patient's age, T stage, N stage, carcinoma stage, drinking status (non-drinker, drinker), smoking status (non-smoker, smoker), and tumor recurrence. Non-smokers were defined as those who smoked ≤10 packs/y, and smokers were defined as those who smoked >10 packs/y. All patients followed up for 2 years. Progression-free survival was defined as the time from registration to local or distant recurrence. Failure was recorded on the occasion of recurrent cancer or if the primary tumor was never wholly gone.

### Cell Culture Conditions

Human laryngeal cancer cell line AMC-HN-8 cells were obtained from the Asian Medical Center, Ulsan University College of Medicine approximately 8 years ago. The cells were cultured in RPMI-1640 (Gibco, CA, U.S.A.), supplemented with streptomycin (100 μg/ml; Beyotime), penicillin (100 units/ml; Beyotime, China) and 10% fetal bovine serum (Gibco), in a 5% CO_2_ incubator at 37°C.

### Establishment of Stable Cell Lines With Overexpression of BCl11A

The lentiviral vectors expressing the Bcl11A-FLAG sequence (Bcl11A-OE) and the NCs were provided by Hanyin Co. (Shanghai). The primers were as follows:

Bcl11a-F: 5′ GACTAGTGCCACCATGTCTCGCCGCAAGCAAGG3′; Bcl11a-R: 5′ CGGGATCCTCACTTGTCGTCATCGTCCTTGTAGTCGAACTTAAGGGCTCTCGAGCTT-3′. Amplified flag-tagged Bcl11a was cloned into a pHY-LV-OE2.2 vector. Recombinant lentiviruses (Bcl11A-OE and NC) (Hanyin Co.) were then arranged and titered to 10^9^ TU/ml. To establish stable cell lines, cells were plated in six-well plates and infected with the virus and polybrene the next day. Positive clones were then incubated with puromycin for 14 d. Bcl11A overexpression was verified by Western blot and qRT-PCR analyses.

### Cell Proliferation Assays

The proliferation effects of AMC-HN-8-NC and AMC-HN-8-BCL11A cells were measured by CCK-8 assay. The cells were plated into 96-well plates, with 100 μl (1 × 10^4^/ml) in each well in the exponential growth phase. The cells were then placed in an incubator at 37°C for 5 d, then 10 μl CCK-8 was added to every well then incubated for 1 h. The absorbance of every unit was assessed at 450 and 650 nm using a standard enzyme-linked immunosorbant assay. The absorbance values at A450 and A650 were calculated, and the results were measured in triplicate.

### Colony Formation Assay

Cells were plated at 800 cells per well in six-well plates (*n* = 2). After 15 d of culture, the cells were stained with crystal violet, photographed and scored.

### Transwell Migration Assays

In the transwell migration assays, 8.0 μm pore polyethylene terephthalate track-etched membrane cell culture inserts (FALCON) were used. A total volume of 600 μl of RPMI-1640 medium (Gibco), supplemented with 20% serum, was loaded into the lower chamber of a 24-well plate. Then, 1 × 10^5^ cells in 100 μl of medium were added to the upper chamber, and the cells were allowed to migrate for 24 h at 37°C. Five random fields per insert were imaged at ×200 magnification. The migrated cells were calculated by ImageJ (NIH Image, NIH).

### Invasion Assays

In the invasion assays, 8 μm transwells were coated with 1 mg/ml of Matrigel (BD Pharmingen). The medium (100 μl) containing the cells (2 × 10^5^) was added to the higher unit, and the cells were allowed to migrate for 24 h in a 37°C incubator. All the experiments were performed in triplicate. The rest of the experimental methods were the same as those used in the migration assays.

### Investigation of Cell Cycle Phase Using Flow Cytometry

The AMC-HN-8 cells were trypsinized, washed with PBS and then fixed by icy 70% ethanol for 24 h. The cells were stained with 1 ml of PI (0.1 mg/ml with 0.1% Triton X-100; Sangon Biotech) and cultured in the dark for half an hour. The cells were evaluated using a flow cytometer (FACSCalibur, Becton-Dickinson).

### Immunochemistry

The tumor tissues were dissected, fixed and embedded in paraffin. The tissue microarray analysis (TMA) was completed with tumors by using multiple representative 1 mm cores from each tumor. Immunohistochemistry staining with haematoxylin/eosin (H&E) of 4–5 μm paraffin sections was performed. The primary antibodies were mouse monoclonal anti-human anti-BCL11A (Clone: EPR 14943-44, 1:100 dilution, Abcam, London, U.K.) and anti-MDM2 (Clone: SMP 14, 1:50 dilution, Abcam). Each tumor was assigned a score based on the staining intensity (no staining = 0, weak staining = 1, moderate staining = 2, and strong staining = 3). The extent of staining was classified as follows: 81–100% = 4, 51–80% = 3, 11–50% = 2, 1–10% = 1, and 0% = 0 (9). The final score was determined by multiplying the intensity scores by the scores for the positively stained cells, with a maximum score of 12 and a minimum score of 0. Scores ≥ 6 were categorized as high expression, and scores lower than this were classified as low expression. The numbers of positively stained BCL11A and MDM2 cells were estimated by two independent researchers who were blinded to the clinical characteristics.

### qRT-PCR Assays

Whole RNA was extracted by TRIzol (Invitrogen, CA, U.S.A.). The total RNA (1 μg) was used for cDNA synthesis by a reverse transcription kit (Takara, Japan) in a 20 μl reaction mixture containing 5 μM of random hexamers, 0.1 μM of a specific primer and 2.5 μM of an oligo (dT) primer. The assay was executed on an Applied Biosystems 7500 Fast System. The expression levels of the target gene RNAs were standardized to that of GAPDH. The BCL11A primer pair was as follow:F:ACAAACGGAAACAATGCAATGG, R:TTTCATCTCGATTGGTGAAGGG.

### Cancer PathwayFinder PCR Array

The RNA was extracted from the AMC-HN-8-BCL11A cells and AMC-HN-8-NC cells in the exponential growth phase using TRIzol (Invitrogen). The gene expression of 84 genes in nine biological tumorigenesis pathways was measured using the Human Cancer Pathway-Finder RT^2^ Profiler PCR Array (SABiosciences) according to established guidelines and evaluated using the MX3005P Real-Time PCR System (Stratagene). Only genes with 1.5-fold variation or greater were selected for further research. The data were confirmed using a combination of qPCR and Western blot assays.

### Western Blot Analysis

The total cell extracts were processed by denaturing proteins with SDS loading buffer (Beyotime, China). Equivalent amounts of protein were separated by 12% SDS-polyacrylamide gel electrophoresis, transferred to polyvinylidene fluoride membranes (Millipore Corporation, MA, and U.S.A.) and then blocked for 30 min in Tris-buffered saline with Tween 20 (TBST) and 5% non-fat milk. The blots were incubated at 4°C overnight in a series of antibodies diluted in TBST buffer: BCL11A (Clone: EPR 14943-44, Abcam, London, U.K.) and MDM2 primary antibodies (1:5,000), baculoviral IAP repeat containing 3 (BIRC3) antibody (Clone: E 40, 1:1000 dilution, Abcam), β-2-microglobulin (B2M) antibody (Clone: EP2978Y, 1:5000 dilution, Abcam), STMN1 antibody (Clone: EP1573, 1:50000 dilution, Abcam), SNAI2 antibody (ab75629, 1:50000 dilution, Abcam), haem oxygenase 1 (HMOX1) antibody (Clone: HO-1-1, 1:1000 dilution, Abcam), and protein phosphatase 1 regulatory inhibitor subunit 15A (PPP1R15A) antibody (ab 9869, 1:1000 dilution, Abcam). After washing with TBST, the membranes were incubated in secondary antibodies (1:4,000; KPL, MD, USA) and then analyzed by a chemiluminescence detection kit (Thermo Scientific, Inc., NJ, USA).

### Xenograft Studies

The tumor growth of the AMC-HN-8-NC and AMC-HN-8-BCL11A cell lines (1 × 10^6^ cells per cell line) was determined following a subcutaneous injection of cells into the left flank of 6-week-old male nude mice (Department of Laboratory Animal Science, Fudan University), with five animals in each group. The tumor size was analyzed (mm^3^) by means of the formula: 0.5 × (L × W^2^). The tumor specimens were fixed using 4% paraformaldehyde for paraffin embedding. Paraffin sections were stained with H&E according to established guidelines. All animal studies were approved by the Animal Center, Eye Ear Nose and Throat Hospital, Fudan University.

### Statistical Analysis

The associations between BCL11A and MDM2 status and the recorded clinicopathological characteristics were assessed using a two-sample *t*-test, with χ2 tests used for categorical variables. A two-tailed *P*-value of 0.05 or less was considered statistically noteworthy. All the data were investigated by SPSS version 19.0 (IBM, Portsmouth, U.K.).

## Results

### BCL11A Was Highly Expressed in LSCC Patients

**Figure 1:**
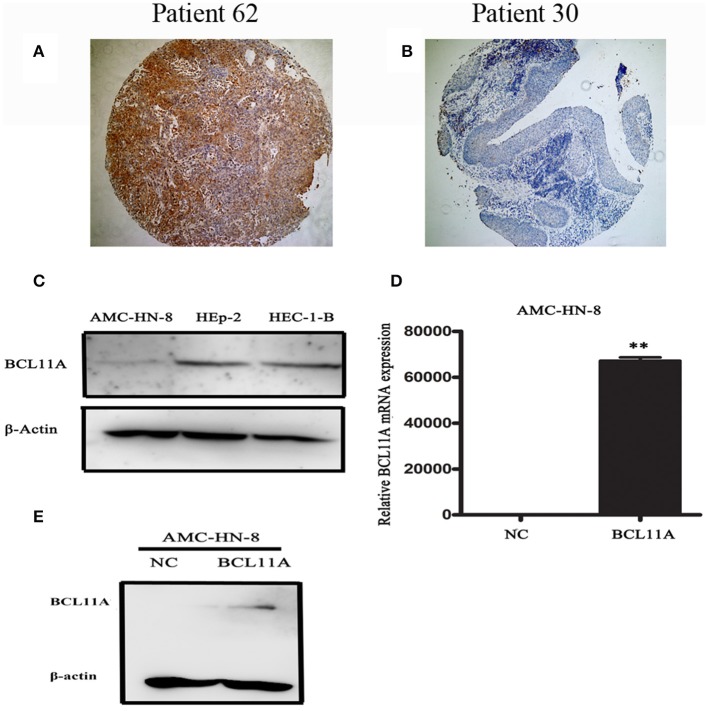
Representative images of BCL11A-positive staining in LSCC cells **(A)** and BCL11A– negative staining in LSCC sections **(B)**. **(C)** A Western blot revealed low expression in the AMC-HN-8 cells. HEC-1-B was used as the positive control for BCL11A expression. **(D,E)** BCL11A overexpression in the AMC-HN-8 cells confirmed by qRT-PCR and Western blot analyses. **P < 0.01.

Of 69 patients enrolled in this study, immunohistological examination revealed that 52 (75.4%) tumors were classified as high BCL11A expression, while 15 (37.5%) paracancer tissues expressed high BCL11A (*p* < 0.01) (Figures 1A,B) (Table 1). Although age, smoking and alcohol did not differ significantly between groups, most of the BCL11A high-expression patients were diagnosed in the advanced clinical N1/N2 lymph node metastasis stage (*p* = 0.023) (Table 2).

**Table 1:** Relationship between BCL11A expression and tissue types.

**Tissue type**	**No**.	**Low**	**High (%)**	**OR (95% CI)**	***P*-value**
Paracancer tissue	40	25	15 (37.5%)		
Carcinoma	69	17	52 (75.4%)	5.1 (2.20–11.84)	<0.01

**Table 2:** Relationship between BCL11A expression and clinicopathological characteristics.

**Parameter**	**No**.	**Low (%)**	**High (%)**	**OR (95% CI)**	***P*-value**
**AGE**					
<60	19	5 (26.3%)	14 (73.7%)	1.13 (0.34–3.79)	0.842
≥60	50	12 (24%)	38 (76%)		
**SMOKING**					
Never	20	3 (15%)	17 (85%)	0.44 (0.11–1.75)	0.235
Former or current	49	14 (28.6%)	35 (71.4%)		
**ALCOHOL**					
Never	34	5 (14.7%)	29 (85.3%)	0.33 (0.10–1.07)	0.093
Former or current	35	12 (34.3%)	23 (65.7%)		
**LYMPHATIC METASTASIS**					
N0	31	12 (38.7%)	19 (61.3%)	4.2 (1.27–12.65)	0.023
N1–2	38	5 (13.2%)	33 (86.8%)		
**PATHOLOGICAL STAGE**					
Initial (I + II)	14	6 (42.9%)	8 (57.1%)	3 (0.86–10.45)	0.076
Advanced (III+IV)	55	11 (20%)	44 (80%)		
**SURVIVAL STATUS** (17 PATIENTS WERE LOST TO FOLLOW)					
Survivors	36	16 (44.4%)	20 (55.6%)	12 (1.43–100.8)	0.009
Deceased or recurrence	16	1(6.3%)	15 (93.7%)		

In accordance with previous studies, high expression of BCL11A was associated with significantly higher loco-regional recurrence or mortality (hazard ratio: 12; 95% CI, 1.43–100.8, *P* = 0.009) (Table 2).

### High BCL11A Levels Promoted LSCC Development

BCL11A, a transcription factor and component of the BRAF complex, has been shown to be involved in regulating cell proliferation, apoptosis and transformation (10). We next investigated the cellular and molecular mechanisms of high levels of BCL11A in LSCC. We started by testing whether BCL11A overexpression affects LSCC cells *in vitro*. We examined the LSCC cell line AMC-HN-8 and found that it expressed low levels of BCL11A (Figure 1C). The AMC-HN-8 cell line has been extensively used to study LSCC (11), and we chose to test BCL11A overexpression in AMC-HN-8 cells. The effects of BCL11A overexpression (Figures 1D,E) were confirmed at the RNA and protein levels.

#### BCL11A Promoted LSCC Cell Proliferation *in vitro*

**Figure 2:**
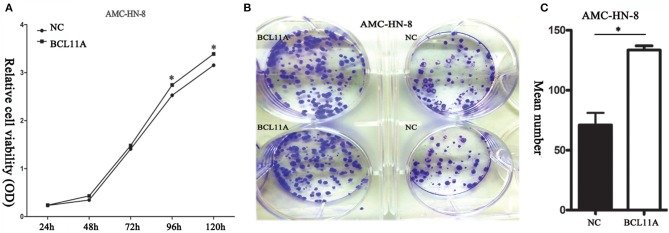
**(A)** BCL11A overexpression increased AMC-HN-8 cell proliferation. **(B)** Overexpression of BCL11A in the AMC-HN-8 cells significantly increased their colony-forming abilities. **(C)** Quantitative comparison of the colonies formed from the parental AMC-HN-8 cells (NC) and the BCL11A overexpression (BCL11A) ones. *P < 0.05.

BCL11A overexpression promoted proliferation in AMC-HN-8 cells (Figure 2A). Furthermore, the BCL11A-overexpressing AMC-HN-8 cells formed more colonies than did the parental cells (Figures 2B,C). These results revealed that one important function of BCL11A is to promote LSCC cell proliferation.

#### Effects of BCL11A Expression on LSCC Cell Migration and Invasion

**Figure 3:**
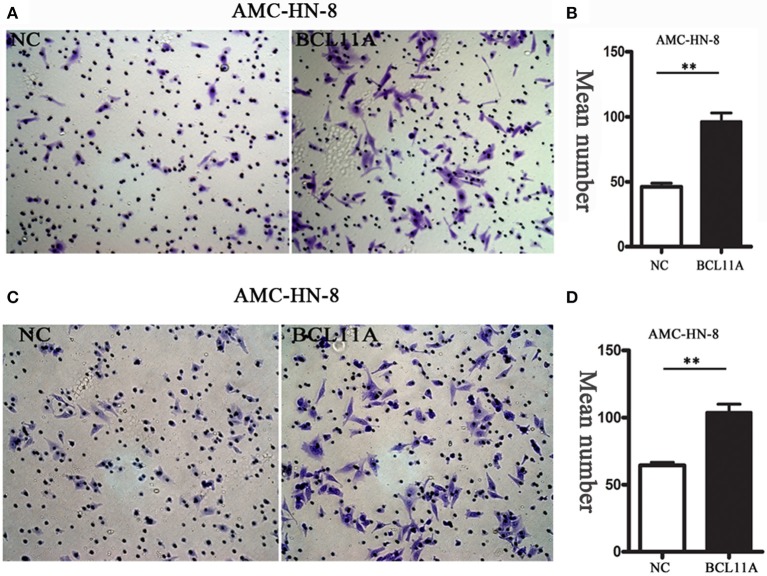
The migration/ invasion ability of LSCC cells with overexpression in the transwell assay. **(A)** Increased migration capability of BCL11A-overexpression AMC-HN-8 cells detected by the transwell assay. Images showing the migrated AMC-HN-8 cells on the lower surface of the transwell membranes. Magnification ×200. **(B)** The numbers of migrated AMC-HN-8 cells in five random fields under the microscope (mean ± SD, *n* = 5). **(C)** Increased invasion capability of BCL11A-overexpression AMC-HN-8 cells. Images showing the invaded BCL11A-overexpresing AMC-HN-8 cells on lower surface of the transwell membranes in different groups. Magnification ×200. **(D)** The number of invaded BCL11A-overexpressing AMC-HN-8 in five random fields under the microscope (mean ± SD, *n* = 5). **P < 0.01.

We next investigated the effect of BCL11A on cell migration and invasion in LSCC cell lines. Transwell assays showed that overexpression of BCL11A significantly increased the migration and invasion capacity of AMC-HN-8 cells (Figures 3A–D). The results indicated that overexpression of BCL11A seemed to increase the migration and invasion of these cells.

#### Change in the Chemosensitivity of Cisplatin After BCL11A Overexpression

**Figure 4:**
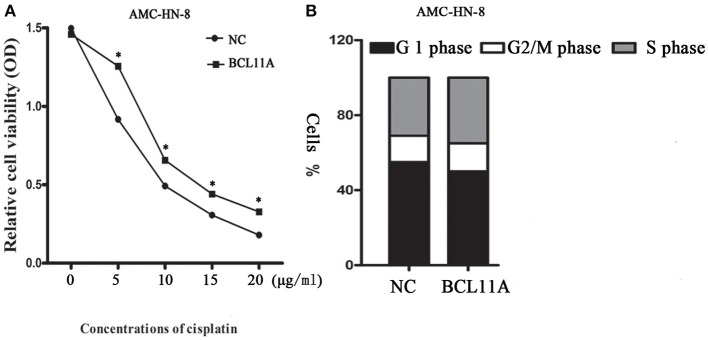
**(A)** Influence of different concentrations of cisplatin on the cytotoxicity of negative controls (NC) AMC-HN-8 and overexpression BCL11A AMC-HN-8 cell strains. **(B)** Cell cycle investigation, showing that overexpression did not affect cell cycle kinetics in the two cell lines. The data are the mean ± s.d. (*n* = 3).*P < 0.05.

We next addressed whether changes in BCL11A in AMC-HN-8 cells could have an impact on chemosensitivity to cisplatin. Overexpression of BCL11A in AMC-HN-8 cells significantly increased their survival or inhibited chemosensitivity to cisplatin (Figure 4A). The results show that BCL11A-high tumors could be more resistant to chemotherapies such as cisplatin.

#### Impact of BCL11A on the Cell Cycle

Overexpression of BCL11A had no significant effect on cell cycle kinetics (Figure 4B).

#### BCL11A Promoted LSCC Growth *in vivo*

**Figure 5:**
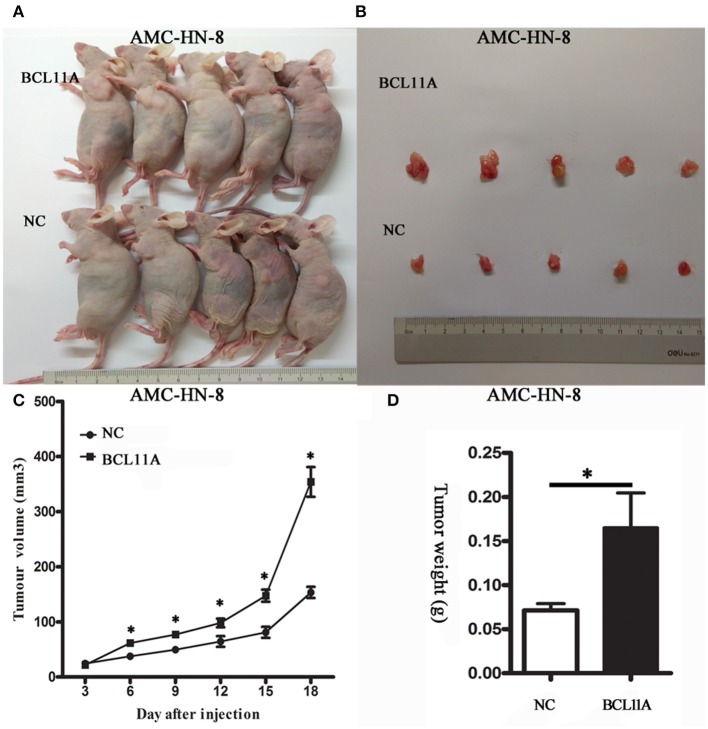
BCL11A regulated AMC-HN-8 cell growth *in vivo*. **(A)** Images of mice with xenograft tumors of BCL11A-overexpression AMC-HN-8 cells and the control cells (NC); **(B)** Image of tumors isolated from nude mice. **(C)** Growth curves of the xenografts in the BCL11A-overexpression AMC-HN-8 cell group and control cells (NC) *(n* = 5, mean ± SEM). **(D)** Comparison of the weight of the xenografts in the BCL11A-overexpression AMC-HN-8 cells and control cells (NC) harvested 18 d after injection of the cells (*n* = 5, mean ± SEM). *P < 0.05.

The substantial effects of BCL11A expression changes on cell proliferation, cell invasion and chemo-sensitivity prompted us to investigate the consequences of BCL11A expression changes *in vivo*. LSCC cells overexpressing BCL11A were injected into immunocompromised recipient mice to induce tumor development. The tumors in the mice injected with the BCL11A-overexpressing AMC-HN-8 cells were larger and heavier (*P* < 0.05 for both; Figures 5A–D). These *in vivo* results demonstrate that BCL11A has important functions for LSCC *in vivo* tumor development.

#### Correlation of BCL11A and MDM2 in LSCC

**Figure 6:**
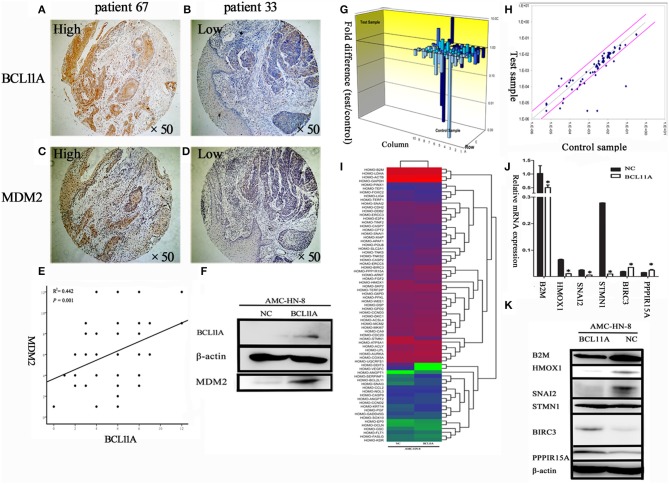
**(A–D)** Co-expression of BCL11A and MDM2 in some LSCCs, as shown by immunohistochemistry. **(E)** Linear regression analysis of BCL11A vs. MDM2 expression levels (immunohistochemistry scores in the same TMA). **(F)** The Western blot showed that MDM2 was up-regulated in the BCL11A overexpression AMC-HN-8 cell line at the protein level. **(G)** PCR microarray investigation of human cancer pathway genes in the BCL11A-overexpression AMC-HN-8 cells. 3D profile graph displays the fold change in the expression of each gene in the BCL11A-overexpression AMC-HN-8 cells vs. that of the control cells. Columns pointing up (with z-axis values > 1) indicate up-regulation of gene expression, and the columns pointing down (with z-axis values < 1) denote down-regulation of gene expression. **(H)** The corresponding scatter plots display the validity of the test and expression level of every gene in the BCL11A-overexpression AMC-HN-8 cells vs. that of the control cells (*n* = 3). **(I)** Heat map of the 84 probe sets in BCL11A-overexpression AMC-HN-8 cells vs. that of the control cells. **(J)** Six selected genes in the BCL11A-overexpression AMC-HN-8 cells showed a significant change at the mRNA level vs. that of the control cells. **(K)** Western blot verification of the gene expression changes. *P < 0.05.

It has been shown in breast cancer cells that BCL11A positively regulates MDM2 expression (12). To evaluate the potential association of BCL11A and MDM2 in LSCC, we evaluated BCL11A and MDM2 co-expression by immunochemistry in the same tissue microarrays (TMA) from 69 LSCC patients (Figures 6A–D). Each tumor was assigned a BCL11A and MDM2 expression score based on the staining intensity and staining extent. A significant direct correlation was identified between the expression of BCL11A and MDM2 in the samples (*r* = 0.442; *P* = 0.001; Figure 6E). Experimentally, overexpression of BCL11A in AMC-HN-8 cells increased the MDM2 protein level (Figure 6F). Clinically, the LSCC cases that simultaneously expressed high BCL11A and MDM2 levels exhibited high rates of mortality or recurrence compared to the other patient subgroups (11/23 vs. 5/29). These data suggest that one function of BCL11A in LSCC is the regulation of MDM2.

#### Regulation of Tumorigenesis Molecules After BCL11A Overexpression

To identify other molecules or pathways that were regulated by BCL11A in LSCC, the Human Cancer PathwayFinder PCR Array analysis of nine different biological pathways (cell cycle, angiogenesis, cell senescence, DNA damage and repair, apoptosis, epithelial-to-mesenchymal transition, metabolism, hypoxia, telomeres and telomerase) (12) was performed (Figures 6G–I). The results revealed significant expression changes in several genes, including B2M, HMOX1, SNAI2, STMN1, BIRC3, and PPP1R15A in the AMC-HN-8 cells overexpressing BCL11A (Figure 6J). The Western blot analysis verified the expression changes of these proteins, except for STMN1 (Figure 6K). The altered gene expression of these molecules could represent additional mechanisms underlying the activity of BCL11A in LSCC.

## Discussion

The BCL11A gene, which spans over 102 kb on chromosome 2p16, is associated with various cancers (6, 13). It was initially observed in B-cell chronic lymphocytic leukemia, and it is considered a proto-oncogene of malignant hematological diseases (3, 14). Khaled et al. demonstrated that BCL11A was a new breast tumor gene and an important factor in normal mammary epithelial development (7). Other research indicated that the expression of BCL11A was reduced in bone marrow and brain, lymphoid and fetal liver tissue (15, 16).

BCL11A was also reported to be amplified in lung squamous cell cancer (SCC), with amplification more common among SCC samples of NSCLC without metastases (17). Jiang et al. reported the possible role of BCL11A in identifying and predicting the prognosis of NSCLC patients, particularly those with early-stage SCC. In their study, BCL11A was specifically overexpressed in tumor tissues rather than paracancer tissues. In the present study, BCL11A was also specifically overexpressed at the protein level in LSCC tissues rather than paracancer tissues. These findings are also consistent with the results of Zhang et al. (18), who observed that BCL11A-XL was markedly overexpressed in large cell lung cancer and squamous cell lung cancer.

Khaled et al. reported that knockdown of BCL11A expression in TNBC cells in a mouse model markedly decreased cancer development (7). In this study, BCL11A overexpression in the AMC-HN-8 LSCC cell line increased LSCC proliferation and invasion *in vitro* and promoted the growth of LSCC xenografts *in vivo*. Overexpression of BCL11A had no noteworthy effects on cell cycle kinetics (Figure 4B), which is similar to the results presented by Khaled et al. (7). They also reported that the survival rates of triple-negative breast cancer patients with either copy number gains or high expression of BCL11A were poorer than those of the remainder of the group. In addition to lower survival rates, the patients with copy number gains of BCL11A had a higher rate of metastasis and relapse. In our study, LSCC patients with high expression of BCL11A were significantly more likely to suffer loco-regional recurrence or to become moribund than those with low expression of BCL11A (hazard ratio: 12, *P* = 0.009) (Table 2). Furthermore, the vast majority of patients with high expression of BCL11A were diagnosed with advanced clinical N1/N2 lymphatic metastasis *(P* = 0.023) (Table 2). Previous studies of four other tumor patient data sets reported a similar tendency (19–22). On the other hand, some studies have shown that BCL11A is an independent prognostic factor for both disease-free survival and overall survival (8, 18). The role of BCL11A expression as a biomarker in the clinic requires additional exploration.

It was reported that BCL11A can also upregulate MDM2 expression, which hinders p53 activities (23). These molecular changes happen frequently in solid cancers. Yu et al. reported that Mdm2 was downregulated in Bcl11a-deficient B cells. ChIP tests showed that Bcl11a was bound to Mdm2. Furthermore, overexpressing Mdm2 rescued both apoptosis and proliferation defects caused by Bcl11a knockdown in B cells. In addition, Bcl11a deletion resulted in p53 accumulation in lymphocytes, likely due to reduced Mdm2/Mdm4 (24). In the current study, as shown by the BCL11A and MDM2 immunochemistry scores in the LSCC TMA, there was a significant correlation between BCL11A and MDM2 expression (*r* = 0.442; *P* = 0.001; Figure 6E). In the LSCC cell line, overexpression of BCL11A caused high expression of MDM2 (Figure 6F). However, the relevance of MDM2 expression highly depends on p53 status, and further research will be focused on the relationship between P53 status and MDM2.

We applied the Cancer PathwayFinder PCR array to identify additional genes regulated by BCL11A in LSCC. Among the upregulated genes in the AMC-HN-8-BCL11A cells, some genes are classified as involving apoptosis, DNA damage, and DNA repair; BIRC3 promotes resistance of cancer cells to apoptosis (25, 26). Another gene, PPP1R15A, was previously shown to be involved in apoptosis or cell growth (27). Among the downregulated genes in the AMC-HN-8-BCL11A cells, some genes were associated with the epithelial-to-mesenchymal transition and hypoxia, including B2M, which is a Class I MHC protein that regulates the proliferation of normal cells and carcinoma cells (28). HMOX1, the inducible isoform of the rate-limiting enzyme in haem degradation, neutralizes inflammatory and oxidative injury. HMOX-1 deficiency results in high levels of endothelial damage and prolonged inflammation (29, 30). In addition, SNAI2, which represses E-cadherin (CDH1), was also downregulated. In renal cell tumors, SNAI2-positive renal cell tumors were reported to be associated with lower-stage tumors and a better prognosis than SNAI2-negative tumors (31).

## Conclusions

This study investigated the proto-oncogene BCL11A in LSCC. The results revealed that BCL11A was upregulated in some clinical LSCC tissue samples at the protein level. The statistical analyses showed that BCL11A expression was strongly associated with lymph node status and the survival of LSCC patients. We demonstrated experimentally that BCL11A regulated cell proliferation, cell invasion/migration and chemosensitivity in LSCC cells *in vitro* and in LSCC xenografts *in vivo*. BCL11A also upregulated the expression of MDM2, which is frequently co-expressed in LSCC.

## Data Availability Statement

The raw data supporting the conclusions of this article will be made available by the authors, without undue reservation, to any qualified researcher.

## Ethics Statement

The studies involving human participants were reviewed and approved by the Ethics Committee of Fudan University. The patients/participants provided their written informed consent to participate in this study. The animal study was reviewed and approved by the Ethics Committee of Fudan University.

## Author's Note

We demonstrated that BCL11A expression was strongly associated with lymph node status and the survival of LSCC patients and BCL11A regulated cell proliferation, cell invasion/migration and chemosensitivity in LSCC cells *in vitro* and LSCC xenografts *in vivo*. BCL11A also up-regulated the expression of MDM2, which is frequently co-expressed in LSCC. Our results uncovered important functions of BCL11A in LSCC and identify BCL11A as a prognostic biomarker and potential therapeutic target in LSCC.

## Author Contributions

LT and L-ML designed and conceived the experiments. JZ, LiaZ, DZ, W-JT, DT, X-LS, and YueY collected samples. JZ, LinZ, FL, and YonY completed the experiment. JZ, LiaZ, and PL analyzed the experimental data. JZ and DZ finished the paper.

### Conflict of Interest

The authors declare that the research was conducted in the absence of any commercial or financial relationships that could be construed as a potential conflict of interest.
